# Genetic characterization and pathogenicity analysis of three porcine epidemic diarrhea virus strains isolated from North China

**DOI:** 10.1186/s13567-025-01554-4

**Published:** 2025-06-14

**Authors:** Ying Liu, Jinghui Fan, Wenyuan Gu, Yunhuan Zhao, Shuai Zhang, Yuzhu Zuo

**Affiliations:** 1 College of Veterinary Medicine, Hebei Agricultural University, Baoding, China; 2 Hebei Animal Disease Control Center, Shijiazhuang, China; 3 Hebei Veterinary Biotechnology Innovation Center, Baoding, China

**Keywords:** PEDV, S protein, molecular structure characterization, evolution trend, pathogenic analysis

## Abstract

Porcine epidemic diarrhea (PED) is a highly contagious intestinal disease owing to porcine epidemic diarrhea virus (PEDV) infection. It is extremely detrimental to newborn piglets and has caused huge economic losses to the global pig industry. In this study, three PEDV strains of G2a PEDV-WF/2023, G2b PEDV-SX/2024 and PEDV-HS/2024 were successfully isolated from small intestine tissue samples with the analysis of their molecular structure characteristics, genetic characteristics and pathogenicity. Notably, these three PEDV strains had multiple unique aa mutations and extensive N-glycosylation in the D0 region, S1-NTD, COE epitope and SS6, respectively. Therefore, their structures were different compared to CV777 and PT-P5 strains. Furthermore, all the three PEDV strains caused severe clinical symptoms in 1-day-old piglets after infection. Among them, G2a PEDV-WF/2023 was the most detrimental to piglets, with highly levels of viral RNA in vivo. In contrast, PEDV-HS/2024 showed relatively weak pathogenicity to piglets, but it also caused the death of piglets. It might be attributed to the occurrence of individual mutations consistent with the amino acid sequence of G1b subtype in PEDV-HS/2024 strain. Findings in this study allow us to confirm that the G2a PEDV-WF/2023 strain is currently one of the most harmful epidemic strains to piglets. This study may benefit our understanding of the molecular structure characteristics, evolution trend and transmission dynamics of the epidemic strains in China. Moreover, it may provide potential reference for formulating more targeted PEDV vaccines, preventing and controlling this infection, and further curbing the cross-species spread of PEDV.

**Supplementary Information:**

The online version contains supplementary material available at 10.1186/s13567-025-01554-4.

## Introduction

Coronaviruses, named for their crown-like spikes on its surface, are a type of single-stranded positive-sense RNA virus, which is the largest RNA genome known currently [1]. The coronavirus family includes Alpha-coronavirus, Beta-coronavirus, Gamma-coronavirus and Delta-coronavirus [2]. Porcine epidemic diarrhea virus (PEDV) is a member of the genus Alpha-coronavirus in the family Coronaviridae, which may cause severe watery diarrhea, vomiting, dehydration, etc. [3]. It is especially detrimental in neonatal piglets, with mortality rates of up to 80–100% [4]. PEDV spread globally since its first detection in Europe in 1971 [5], resulting in significant economic losses. In the early 2010 s, PEDV swept through China and Southeast Asian countries, causing a large-scale PED outbreak, especially in 2013, exerting a huge impact on the pig industry [6, 7]. So far, PEDV is still one of the problems plaguing the global pig industry.

PEDV genome has a full length of approximately 28 kb and an envelope structure [8], encoding multiple structural and non-structural proteins. Its structural proteins mainly include spike protein (S protein), membrane protein (M protein), envelope protein (E protein), nucleocapsid protein (N protein) and ORF3 (accessory protein) [9].

The S protein is generally considered to be a key factor in the interaction between viruses and host cells [10], consisting of two subunits (S1 and S2). Specifically, the S1 subunit is responsible for the cell receptor recognition of the virus [11]; while the fusion peptide and α-helix structure in the S2 subunit will promote the membrane fusion of the virus and cells during virus invasion [12, 13]. Currently known neutralizing antibody epitopes include the NTD/S10 region located at the N-terminus of the S protein [1]; the COE region located on the surface of the S protein [14]; as well as linear epitopes that can be directly recognized by antibodies [15]. There are approximately 25 potential glycosylation sites in the PEDV S protein. It can affect both the stability and invasion of the virus [16]. PEDV divides the virus into two genotypes based on the S gene, i.e., G1 and G2. G1 genotype includes two subtypes, G1a and G1b, which is the genotype recognized globally earlier. G2 genotype is further subdivided into G2a and G2b subtypes. G2 genotype, with a higher pathogenicity, is currently the main prevalent genotype in China and some other countries [7, 17]. The emergence of S-INDEL genotype strains may change the S1 region structurally [18], with specific insertions and deletions in the S gene [19]. These variations may have arisen through natural selection and adaptive evolution, posing potential challenges to the development of PEDV vaccines [20]. In addition, ORF3 protein has ion channel activity and exhibits ion channel function in different cell models [10, 21]. There are also significant differences in the genetic makeup between wild-type and attenuated PEDV [22], inducing different pathogenicity and immune evasion strategies possibly [7].

At present, with continuous emergence of multiple mutant strains of PEDV, the diversity of genetic variation of the virus allows it to evade the immune protection of existing vaccines. Consequently, existing commercial PED vaccines currently are insufficient in protecting against popular strains, compromising the effectiveness of the vaccine [23]. Therefore, this study successfully isolated a PEDV G2a strain, PEDV-WF/2023, and two PEDV G2b strains, PEDV-SX/2024 and PEDV-HS/2024, from diarrheal fecal samples in North China. This study further analyzed the differences in genomic characteristics, viral structure and pathogenicity of these three strains. These data are particularly important for understanding the structural characteristics, molecular characteristics, evolution trends, transmission dynamics, pathogenicity, and other aspects of the currently popular highly pathogenic strains. Based on the unique molecular characteristics of different genotypes, it can also be used for more targeted prevention and control of different genotypic strains of PEDV. In addition, it may provide potential reference for formulating effective prevention and control strategies, and also theoretical basis for the development of new vaccines and treatment options to curb the spread of PEDV in pigs and other species (especially in humans).

## Materials and methods

### Sample collection

This study collected 18 clinical samples (diarrhea feces and small intestine tissues) of dead piglets in North China totally. The processed samples were centrifuged at 8000 rpm for 5 min, followed by the collection of the supernatant for isolation and identification of the virus.

### Isolation and cultivation of viruses

Vero-CCL-81 cells preserved by our laboratory were cultured in an incubator at 37 °C and 5% CO_2_, using Basic Dulbecco’s Modified Eagle’s Medium (DMEM; Gibco) supplemented with 10% (v/v) fetal bovine serum (FBS; Excel), 100 IU/mL penicillin, and 10 µg/mL streptomycin (Solarbio). Before infection, 1 mL of fivefold diluted virus supernatant was added firstly to trypsin with a final concentration of 10 μg/mL for 1 h of incubation in a 5% CO_2_ incubator at 37 °C for activation. Then, the activated virus liquid was added for another 3 h of incubation at 37 °C. Subsequently, trypsin containing a final concentration of 7 μg/mL was added after discarding the virus liquid in the cell culture flask. DMEM serum-free medium was used for culturing and propagating viruses.

### RT-PCR and RT-qPCR detection

Total RNA was extracted from 250 μL of virus supernatant sample using Trizol reagent (TaKaRa, Dalian, China), followed by reverse transcription using BioTR High Sensitivity cDNA First Strand Synthesis Kit (BIOER, Hangzhou, China) Generate cDNA. PEDV M gene and N gene were separately identified by RT-PCR and RT-qPCR to monitor the proliferation of the three isolates on Vero cells. The specific amplification primers of PEDV M gene and N gene are shown in Table 1.

**Table 1:** **Primer sequences used in this study**

Primer ID	Sequence (5′−3′)	Primer length (bp)
PEDV-M-F	TCAGTATGGCCATTACAAGTACTCT	518
PEDV-M-R	TAGTCGCCGTGTTTTGACCGGACAT	
qPEDV-N-F	CATTGGCGAAAACCCTGACAAGCTT	231
qPEDV-N-R	TCAGGCTATGCCCAGATCGCCAGTT	
PEDV-S1-F	ATGACGCCATTTGTGGCTTATC	2362
PEDV-S1-R	GCCACACTGAGATGGGACA	
PEDV-S2-F	TGGCAGAATTCGCTACGTGC	1941
PEDV-S2-R	TGACGACTGTGTCAATCGTGT	
PEDV-ORF3-F	CTAGTGTTCTGCTGCATTTC	885
PEDV-ORF3-R	CAATTGGACGAAGGTAATGC	

### Indirect immunofluorescence (IFA)

The well-grown Vero cells were infected with three isolates (MOI = 0.1), respectively, after inoculation into 96-well-plates. After 3 d of culture, cells were incubated with pre-cooled 4% paraformaldehyde, permeabilized with 0.5% TritonX-100 at room temperature for 10 min, washed three times with phosphate-buffered saline (PBS), and blocked by 5% Bovine Serum Albumin (BSA) at 37 °C for 1 h. PEDV-positive serum was used as primary antibody (1:50) and incubated overnight at 4 °C, after which it was incubated with goat anti-swine IgG antibody (Biodragon, China, 1:250) for 1 h in dark. After that, 4′,6-Diamidino-2-phenylindole dihydrochloride (DAPI; Solarbio, China) was added to stain the cell nuclei for 5 min in dark. These samples were photographed using a fluorescence microscope (Zeiss, Oberkochen, Germany), with corresponding results recorded.

### PEDV virus titer and growth kinetics

The well-grown Vero cells were inoculated into 96-well-plates for virus titer detection, and then the processed cells were infected with tenfold multiplicity dilution of the three PEDV strains, with 6 wells repeated for each sample. Three days after inoculation, this experiment observed the cytopathic effects (CPE) of the cells, and calculated the half tissue culture infection dose (TCID_50_/mL) using the Reed-Muench method.

Inoculation of the three PEDV isolates into Vero cells was performed at MOI = 0.1 to measure their growth curves. Supernatants were collected at 12, 24, 36, 48, 70, 72, and 96 hpi, and cells were infected to determine their viral titers, which were repeated three times.

### Complete sequence alignment as well as phylogenetic analysis of S and ORF3 genes

Viral RNA extraction and cDNA synthesis were performed as described above. The PEDV S and ORF3 genes were amplified by RT-PCR and cloned into pMD19-T vector. The recombinant DNA bacteria were subjected to sequencing by Shanghai Sangon Biotechnology Co., Ltd. (Shanghai, China) to obtain the complete PEDV S and ORF3 genes sequences. The specific amplification primers of PEDV S and ORF3 genes are shown in Table 1. There were 23 coronavirus sequences of different coronavirus genera and 46 sequences of PEDV reference strains of different genotypes in different countries downloaded from NCBI GenBank. Further comparison was made with the three isolates using MAGE 11 software, and neighbor-joining method was employed to analyze the genetic relationship between strains and construct a genetic evolutionary tree. The evolutionary tree was visualized using Chiplot [24]. Homology analysis was performed using DNAStar MegAlign based on the PEDV S and ORF3 genes of the three isolates and representative strains. Amino acid multiple sequence alignment analyses of the S and ORF3 genes were performed using MAGE 11 and the ESPript [25].

### Structural analysis of the PEDV S protein

Physicochemical properties of the three PEDV isolates were captured using the Expasy-ProtParam [26]. Protein secondary structures were predicted by SOPMA method in NPSA [27]. Protein N-glycosylation sites were predicted through NetNGlyc 1.0 [28]. The structures of PEDV CV777 strain (PDB ID: 6U7K) and PT-P5 strain (PDB ID: 7W6M) were used as templates for predicting and comparing the S protein structures of the three PEDV isolates using SWISS-MODEL [29]. The structures were finally compared and annotated by PyMOL 3.1 [30].

### Piglet challenge experiments

Twelve healthy 1-day-old piglets were purchased from commercial pig farms with no history of PEDV vaccination and PEDV infection. The piglets were randomly divided into 4 groups, with 3 piglets in each group. The piglets were placed in separated rooms for rearing at 30–35 ℃, with access to milk each 6 h every day. Three groups of piglets were orally infected with PEDV-WF/2023, PEDV-SX/2024 and PEDV-HS/2024 strains at doses of 10^5^TCID_50_/mL (0.5 mL/piglet), respectively. The other group was set as the control group, and the simulated challenge group received DMEM (0.5 mL/piglet) orally. After the virus challenge, this study regularly monitored the weight, survival rate and clinical manifestations of the virus infection, such as mental state, appetite, diarrhea, vomiting, etc., of piglets in each group every day. The diarrhea of piglets was scored simultaneously (0 point: normal feces; 1 point: pasty feces; 2 points: semi-fluid feces; and 3 points: watery feces) [4, 31]. Viral RNA shedding was further assessed by RT-qPCR after the collection of anal swabs daily from each group. After blood collection from the anterior vena cava of the piglets every other day, RT-qPCR method was also used to evaluate the PEDV virus content.

### Organ index and viral load test

When piglets died or at the end of the experiment, all piglets were euthanized for collecting and weighing intestinal tissues and important tissues such as heart, liver, spleen, lungs and kidneys. The organ index was calculated by the equation of Organ index (%) = organ weight/piglet body weight. A portion of the intestinal tissues was collected from the severely diseased areas and ground into homogenate with saline, and then stored at −80 °C for the detection of PEDV virus in each group.

### Histopathological analysis

A portion of jejunal tissue (about 1 cm) was collected at room temperature, fixed with 4% paraformaldehyde. Then, paraffin-embedded tissue sections of 5 μm in thickness were prepared and stained with hematoxylin–eosin (H&E) for histopathological analysis. The animal treatment protocol of this study was approved by the Animal Ethics Committee of Hebei Agricultural University.

### Statistical analysis

All experimental data were analyzed by t-test or one-way analysis of variance in GraphPad Prism 9.0 with three replicates for each. Data were shown as mean ± standard error, and differences were defined as significant when *P* < 0.05.

## Results

### Virus isolation and identification

**Figure 1:**
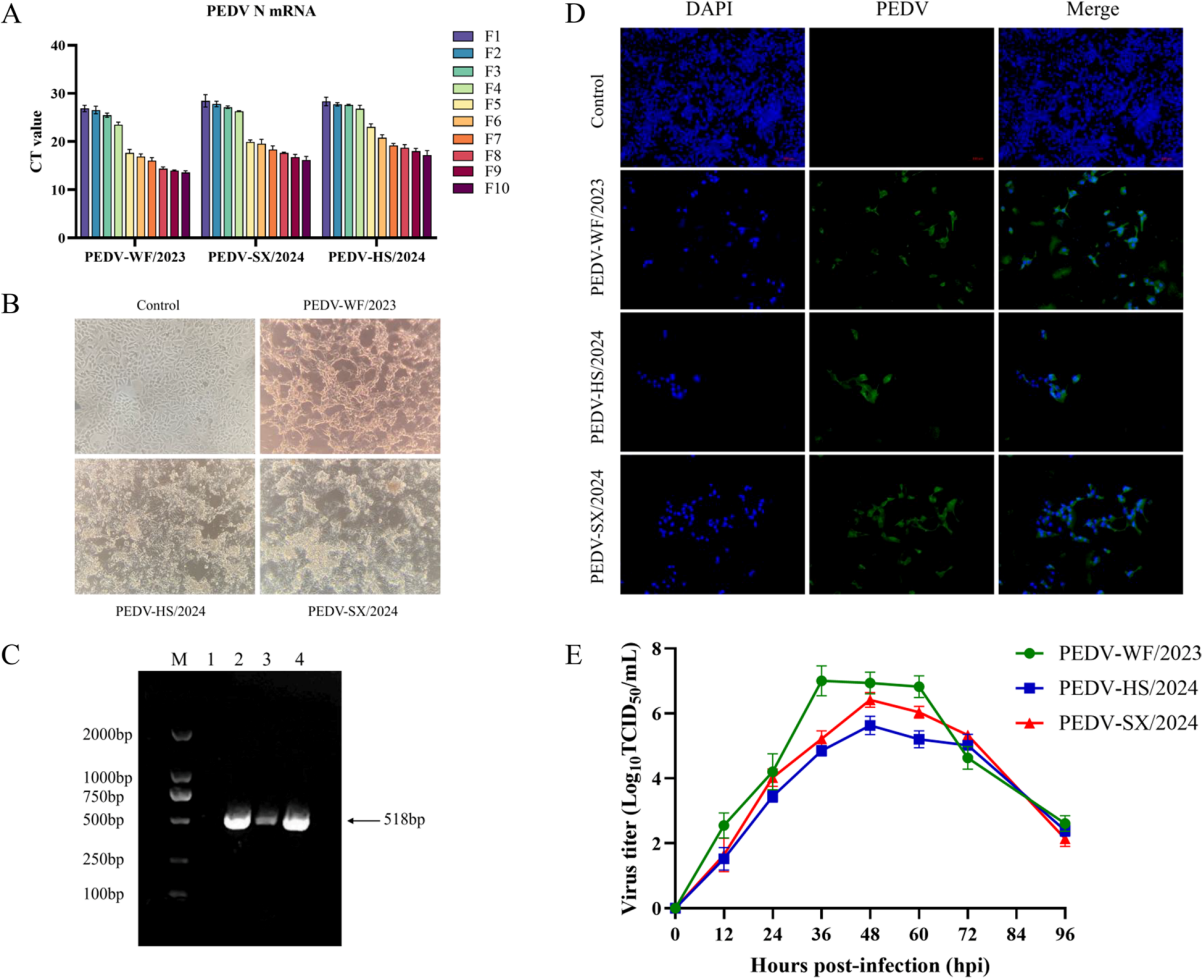
**Isolation and characterization of the PEDV-WF/2023, PEDV-SX/2024 and PEDV-HS/2024 strains**. **A** RT-qPCR detection method was used to identify the mRNA expression level of N gene of each virus strain from F1 to F10 generations. **B** Cytopathic effects of each isolate under light microscopy. **C** The RT-PCR detection method was used to detect the infection of Vero cells by each isolated strain at the F5 generation for the PEDV-M gene. (M) 2000 Marker; (1) Negative Control. (2) PEDV-WF/2023 strain; (3) PEDV-HS/2024 strain; (4) PEDV-SX/2024 strain. **D** Indirect immunofluorescence identification of each isolated virus strain. **E** Detection of growth kinetics of each isolated virus strain.

In this study, continuous passage in Vero-CCL-81 cells obtained three PEDV isolates, namely PEDV-WF/2023, PEDV-SX/2024 and PEDV-HS/2024. RT-qPCR showed significantly increased mRNA levels of all the three viruses from the fifth passage (F5; Figure 1A). Obvious cell fusion characteristic CPEs were observed at F5 (Figure 1B). The three PEDV strains were all positive by RT-PCR for PEDV M gene, and the target fragment was 518 bp (Figure 1C). The IFA results revealed that PEDV whole virus-specific green fluorescence was located in the cytoplasm, and the nuclei of the cells stained by DAPI displayed blue fluorescence. It was confirmed that the PEDV strain had infected the cells after merging the cytoplasm and nucleus (Figure 1D). The results of the virus growth kinetics test showed that during the replication process of the PEDV-WF/2023 strain on Vero cells, it reached a peak at 36 hpi. At this time, the virus titer was 10^7.16^TCID_50_/mL, after which the virus proliferation level gradually decreased with cell shedding. The virus titers of PEDV-HS/2024 and PEDV-SX/2024 peaked at 48 hpi after virus infection, which were 10^6.41^TCID_50_/mL and 10^5.63^TCID_50_/mL respectively (Figure 1E). Taken together, PEDV-WF/2023, PEDV-SX/2024 and PEDV-HS/2024 were successfully isolated on Vero-CCL-81, respectively, and could be stably transmitted.

### Phylogenetic analysis of coronavirus subfamily

**Figure 2:**
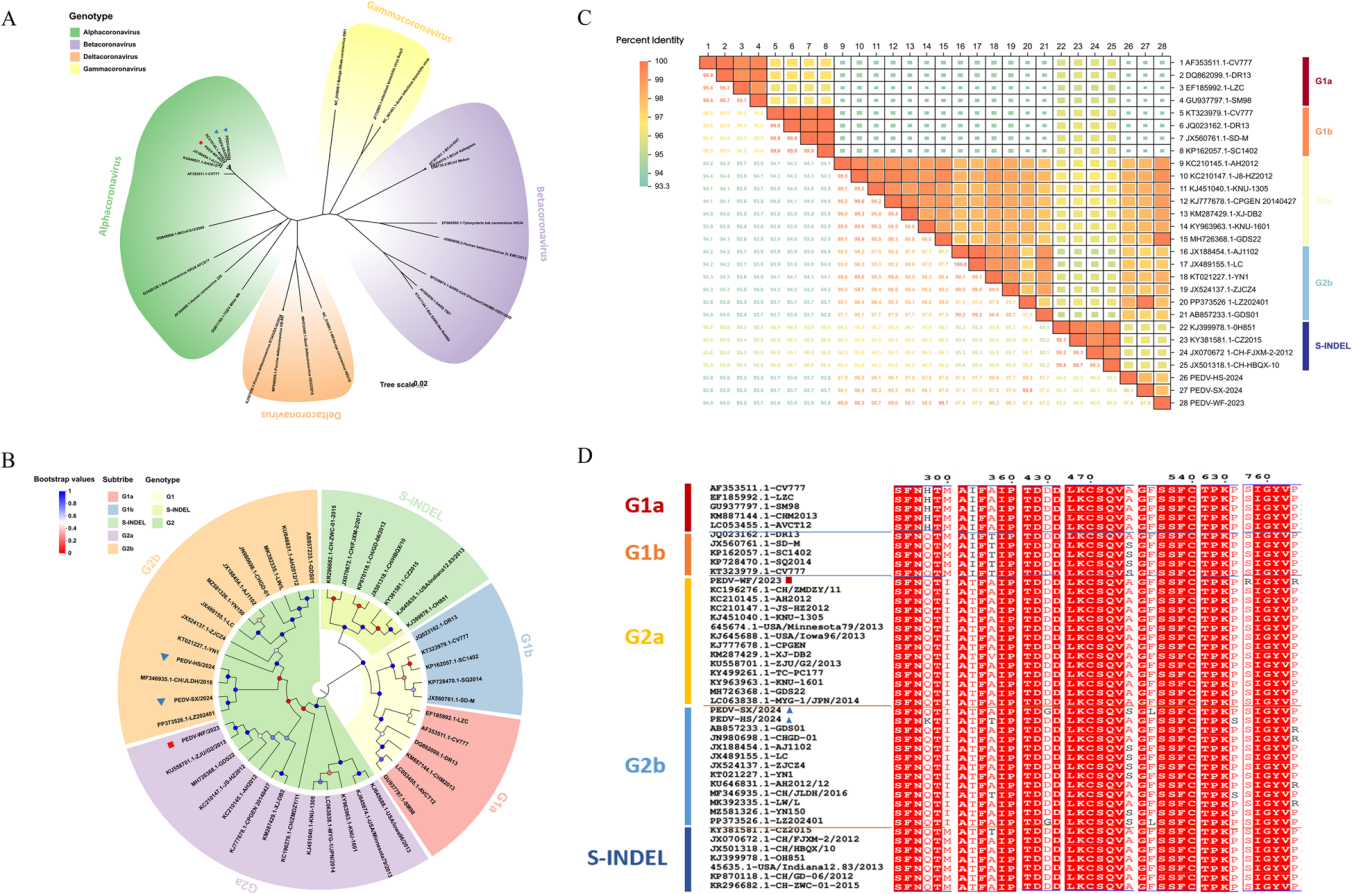
**Phylogenetic tree and genetic characteristics analysis based on S gene**. **A** Coronavirus phylogenetic tree. Different genera are marked with different colors: Alpha-coronavirus (green), Beta-coronavirus (purple), Gamma-coronavirus (yellow) and Delta-coronavirus (orange). **B** Phylogenetic tree of PEDV strains. Different genotypes and subtypes are marked with different colors: G1 (yellow), S-INDEL (grass green), G2 (green) and G1a (red), G1b (blue), S-INDEL (green), G2a (purple), G2b (orange). Phylogenetic trees were constructed with MAGE 11 software using the neighbor-joining method. Bootstrap analysis was set up in 1000 replicates, the color of the dots at the nodes represents the bootstrap values. **C** Heat map of Homology analysis. Different colors represent levels of homology. The change from orange to blue represents the homology from high to low. Among them, the larger the square, the higher the homology, and the smaller the square, the lower the homology. **D** Based on S gene amino acid sequence alignment. Identical amino acid positions are represented by a red background, and mutated amino acids are represented by black fonts. The red square represents the PEDV-WF/2023 strain obtained in this study, and the blue triangle represents the PEDV-SX/2024 and PEDV-HS/2024 strains obtained in this study.

The phylogenetic tree was analyzed based on the S genes of each coronavirus strain (Additional file 1). All coronaviruses were divided into four genera according to the phylogenetic tree, namely Alpha-coronavirus, Beta-coronavirus, Gamma-coronavirus and Delta-coronavirus (Figure 2A). The PEDV strains we studied belonged to Alpha-coronavirus, including BtCoV/512/2005, bat coronavirus HKU8 (BtCoV HKU8 AFCD77), human coronavirus 229 (HCoV-229) and TGEV that were closely related. Moreover, the phylogenetic relationship between PEDV strains and BtCoV/512/2005 was closer than that with TGEV. The highly pathogenic viruses SARS-CoV and SARS-CoV-2, which cause severe respiratory syndrome in humans, belonged to Beta-coronavirus, also including bovine coronavirus, bat coronavirus (BtCoV HKU4) and human coronavirus 2c (HCoV-2c EMC/2012), among others. These Alpha-coronavirus and Beta-coronavirus strains only posed a threat to mammals, whereas Gamma-coronavirus and Delta-coronavirus mainly infected birds, but some of them could also infect mammals, such as porcine delta coronavirus.

### Phylogenetic and genetic characterization of the PEDV S gene

Our study continued after downloading nucleotide sequences of the PEDV S gene reference strains (Additional file 2) from NCBI GenBank. All PEDV strains were classified into three major groups, G1, G2 and S-INDEL (Figure 2B), based on the PEDV S gene phylogenetic tree. The S-INDEL S gene is a recombinant strain based on the G1 genotype (classical strain) and G2 genotype (variant strain). Therefore, S-INDEL was classified as a single genotype in this study. The G1 genotype was divided into two subtypes: G1a and G1b. The G1a subtype (*n* = 6) mainly included the classic strain CV777 and the virulent strain DR13; while the attenuated strains CV777 and DR13 belonged to the G1b subtype (*n* = 5). The S-INDEL genotype was represented by the strain OH851 isolated from the United States in 2014, which was clustered together with six other strains, and was further away from the three strains isolated in this study. The G2 genotype mainly included G2a and G2b subtypes. There were 13 strains of the G2a subtype in the phylogenetic tree. Among them, AH2012 was the representative strain of G2a subtype, which belonging to the same sub-branch with PEDV-WF/2023 isolated in this study and had a close genetic relationship. ZJU/G2a isolated from Zhejiang, China in 2013 was the closest and most closely related to PEDV-WF/2023. The G2b subtype (*n* = 13) with AJ1102 as the representative strain was divided into two distinct sub-branches, and AJ1102 was a currently widely used attenuated vaccine strain and was the same sub-branch as the other 9 strains. The PEDV-SX/2024 and PEDV-HS/2024 strains isolated in this study belonged to the same sub-branch as the LZ202401 strain in Shaanxi, China, and CH/JLDH/2016 in Changchun, Jilin, China, which were most closely related.

Through homology analysis among different strains (Figure 2C), the three PEDV strains isolated here showed homology ranging from 93.3–94% with G1a and G1b subtypes, and 95.9–96% with S-INDEL. PEDV-WF/2023 strain showed a maximum homology of > 99% with the G2a subtype. PEDV- SX/2024 and PEDV-HS/2024 had the highest homology with strain LZ202401 of G2b subtype, 99.8 and 98.3%, respectively, which was consistent with the results of phylogenetic analysis. Collectively, PEDV-WF/2023 isolated in this study was a G2a strain, while PEDV-SX/2024 and PEDV-HS/2024 belonged to the G2b subtype.

Furthermore, this study analyzed aa mutations based on the important structural domains and neutralization epitopes (NTD/S10, COE, SS2, SS6 and 2C10) of the S gene (Figure 2D). It was found that the aa mutations appearing in Q298H, A358T and A474S could perfectly distinguish G1a and G1b subtypes, while the mutation at I356T was a marker for distinguishing G1 and G2 genotypes. However, the characteristics of the aa mutation in the G2a and G2b subtypes were not typical for the S gene. PEDV-WF/2023 had two unique aa mutation sites (S758R and P763R), and PEDV-SX/2024 had three aa mutation sites (D430G, A474S and F536L) that were the same as LZ202401 in the S1-NTD domain and COE epitope respectively. Interestingly, PEDV-HS/2024 had an aa deletion at 1197 aa and the same aa mutation (A358T) as the G1b subtype. Therefore, mutation of 358 aa may be one of the important factors leading to the reduction of PEDV virulence.

### PEDV ORF3 phylogenetic and genetic characterization

**Figure 3:**
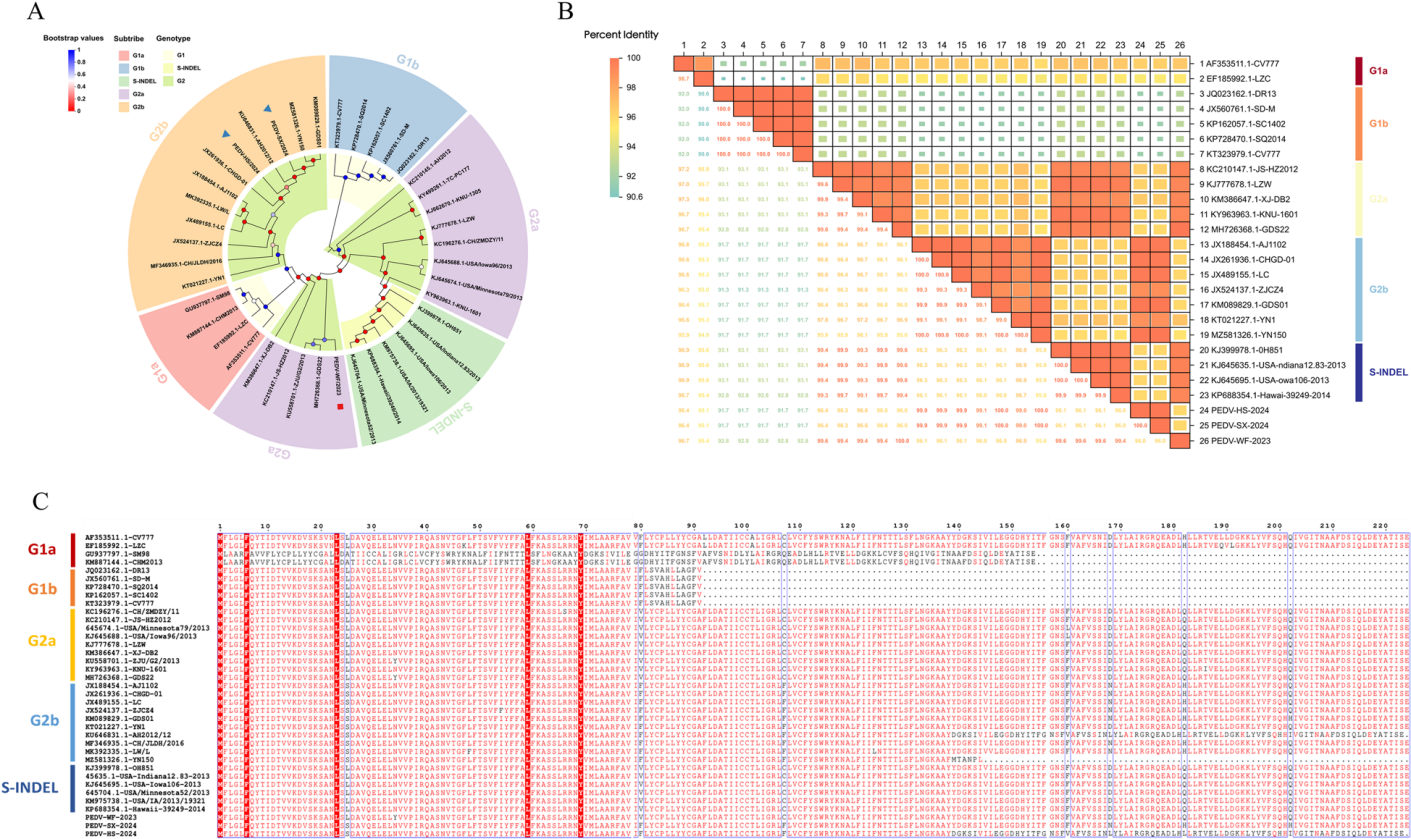
**Phylogenetic tree and genetic characteristics analysis based on ORF3 gene**. **A** Phylogenetic tree of PEDV strains. Different genotypes and subtypes are marked with different colors: G1 (yellow), S-INDEL (grass green), G2 (green) and G1a (red), G1b (blue), S-INDEL (green), G2a (purple), G2b (orange). Phylogenetic trees were constructed with MAGE 11 software using the neighbor-joining method. Bootstrap analysis was set up in 1000 replicates, the color of the dots at the nodes represents the bootstrap values. **B** Heat map of Homology analysis. Different colors represent levels of homology. The change from orange to blue represents the homology from high to low. Among them, the larger the square, the higher the homology, and the smaller the square, the lower the homology. **C** Based on ORF3 gene amino acid sequence alignment. Identical amino acid positions are represented by a red background, and mutated amino acids are represented by black fonts. The red square represents the PEDV-WF/2023 strain obtained in this study, and the blue triangle represents the PEDV-SX/2024 and PEDV-HS/2024 strains obtained in this study.

The phylogenetic relationship and molecular characteristics were analyzed with the ORF3 gene sequences of 37 PEDV strains retrieved from NCBI GenBank (Additional file 3). This part of analysis obtained similar results to the phylogenetic relationship of the S gene (Figure 3A). Interestingly, the phylogenetic tree of the PEDV ORF3 gene indicated that the ORF3 gene of the G2a subtype was intimately associated with the S-INDEL genotype, revealing an extremely high similarity.

Through the analysis of inter-strain ORF3 amino acid homology (Figure 3B), the homology between our studied three strains and the G1 genotype strain ranged between 91 and 96%. Specifically, the PEDV-WF/2023 strain had 99–100% homology with the G2a subtype; while the homology of PEDV-SX/2024 and PEDV-HS/2024 to the G2b subtype was 99–100%. These results were consistent with those of amino acid sequence homology analysis of the S gene, further confirming the genotypes of the three strains isolated in our study. In addition, the homology between G2a and S-INDEL strains also reached 99–100%, supporting the accuracy of clustering the G2a subunit and S-INDEL genotype into the same branch in the ORF3 gene phylogenetic tree.

Next, we performed mutation analysis of amino acids in the whole sequence of ORF3 gene. The G1b subtype had an aa deletion at 92aa-224aa (Figure 3C). Meanwhile, aa mutations appeared at 25aa, 80aa, 107aa, 168aa and 182aa. These five aa mutations could be referenced to perfectly distinguish G2a and G2b subtypes. Altogether, the ORF3 gene has greater amino acid differences, suggesting that it may be more suitable than the S gene for distinguishing G2a and G2b subtypes.

### Structural analysis of the PEDV S protein

In the life cycle of the virus, the S protein acts as a major mediator of viral invasion of host cells, and also possesses important immunogenic properties. It highlights the necessity for analyzing the physicochemical properties, secondary and tertiary structures as well as N-glycosylation of S protein. Firstly, this study compared the physical and chemical properties of the isolates, with G1a type representative strain CV777 (PDB ID: 6U7K) and G2 type representative strain PT-P5 (PDB ID: 7W6M) as the controls. As shown in Table 2, the full-length S protein of PEDV-WF/2023 and PEDV-SX/2024 strains encoded 1386aa, while the PEDV-HS/2024 strain had an amino acid deletion at 1197aa, suggesting that the full-length S protein of this strain encoded 1385aa. The theoretical pI values were 5.35, 5.19 and 5.21, respectively, similar to those of the PT-P5 strain; while the pI value of CV777 was 5.11. The instability index of these three isolates was around 31, and the aliphatic index was above 90, indicating that all of which were hydrophobic proteins.

**Table 2:** **Physicochemical properties of PEDV S protein**

Strains	Number of amino acids	Molecular weight	Theoretical pI	Instability index	Aliphatic index	Grand average of hydropathicity (GRAVY)	Formula
CV777	1383	151352.74	5.11	32.6	93.21	0.123	C_6786_H_10483_N_1751_O_2060_S_56_
PT-P5	1386	151759.9	5.25	31.41	90.19	0.114	C_6817_H_10464_N_1756_O_2061_S_55_
PEDV-WF/2023	1386	152017.34	5.35	31.03	90.75	0.106	C_6826_H_10495_N_1769_O_2057_S_55_
PEDV-SX/2024	1386	151545.85	5.21	32.18	91.45	0.115	C_6793_H_10470_N_1754_O_2061_S_58_
PEDV-HS/2024	1385	151586.77	5.19	31.92	90.18	0.106	C_6796_H_10457_N_1753_O_2063_S_58_

S protein is main structural proteins of PEDV that has a complex secondary structure and is highly important for viral infection. Comparison of CV777 strain and PT-P5 strain with the three PEDV strains (Table 3) revealed that there was no obvious difference in the proportion of secondary structure of the S protein for these strains. The α-helix accounted mainly for about 28%. The proportion of α-helix of CV777 was lower at 27.48%, mainly located in the S2 subunit; while the β-sheets were all above 4%, with little difference. The β-sheet was located almost entirely in the S1 subunit. Furthermore, observation of the 3D structure of the S protein indicated PEDV-WF/2023 and PEDV-SX/2024 had a β-sheet disappeared at 402–403aa compared with PT-P5. PEDV-SX/2024 was also observed to lose a β-sheet at 330–332aa, while PEDV-WF/2023 had a new α-helix at 453aa (data not shown). However, there were shorter amino acids encoded by the mutated secondary structures, and hence these changes in the secondary structure had no significant impact on the structure of the S protein.

**Table 3:** **Secondary structure analysis of PEDV S protein**

Strains	Alpha helix (%)	Extended strand (%)	Beta turn (%)	Random coil (%)
CV777	27.48	27.48	4.41	40.64
PT-P5	27.92	26.91	4.62	40.55
PEDV-WF/2023	28.07	26.7	4.18	41.05
PEDV-SX/2024	28.14	26.12	4.4	41.34
PEDV-HS/2024	27.94	27.29	4.19	40.58

**Figure 4:**
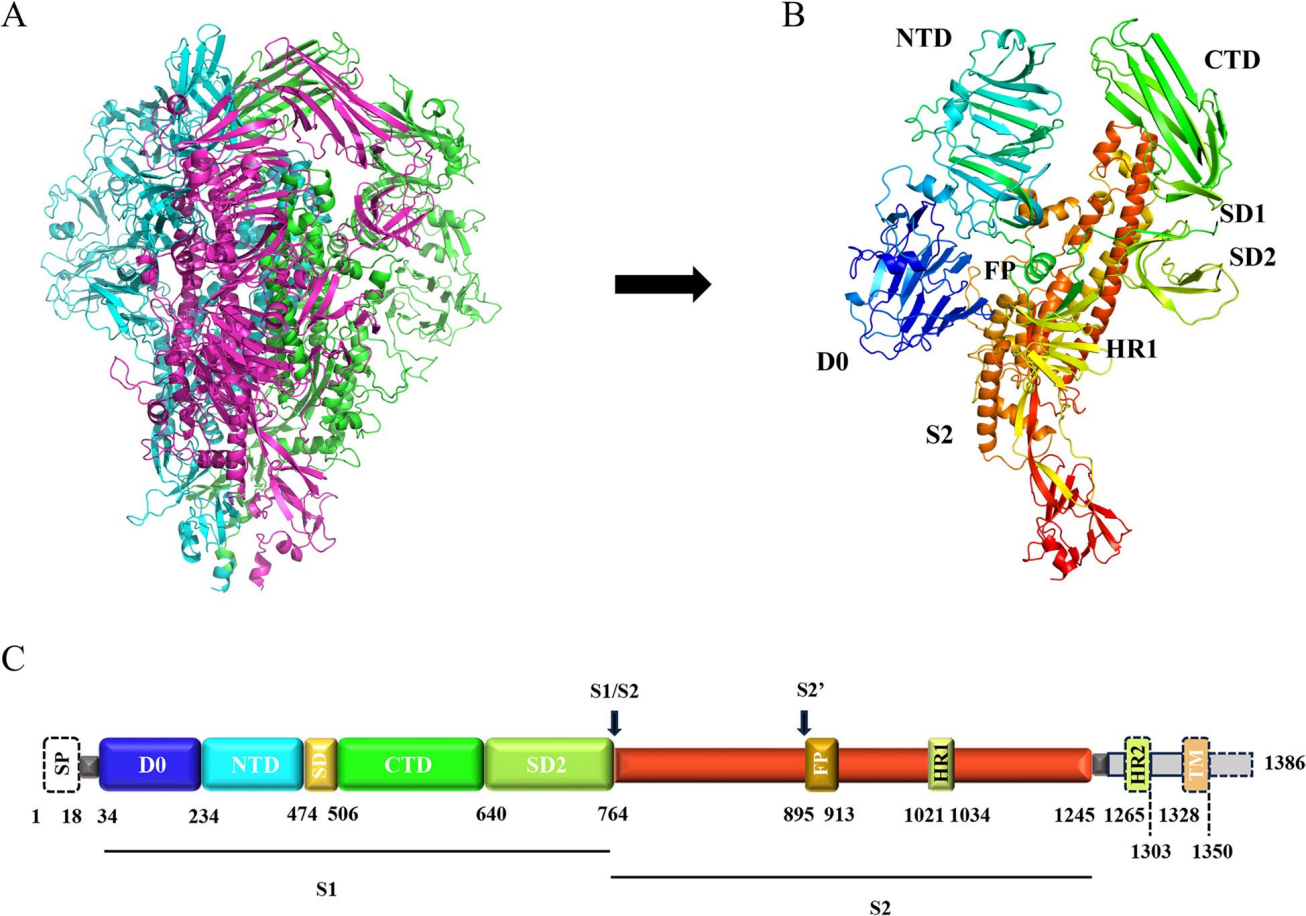
**Schematic structure of PEDV S protein**. **A** Trimer prefusion structure of PEDV S protein. Different colors represent different monomers. **B** Monomer structure of PEDV S protein. Different structural domains are represented by different colors. **C** Genome structure diagram of PEDV S protein. The different colored boxes represent the different domains of the S protein. The same domains in B and C are colored in the same scheme.

This study further analyzed the structural characteristics of the S protein of each strain. Based on the S protein structure of the three isolates, modeling was conducted using SWISS-MODEL. A model of the trimer and monomeric structures of the PEDV S protein are shown in Figures 4A and B, respectively. By integrating Figure 4C, the S protein mainly consisted of two parts, S1 and S2, of which the main structural domains included signal peptide (SP), domain 0 (D0), N-terminal domain of S1 (S1-NTD), subdomain 1 of S1 (SD1), C-terminal domain of S1 (S1-CTD), subdomain 2 of S1 (SD2), fusion peptide (FP), heptad repeat 1 (HR1), heptad repeat 2 (HR2), and transmembrane domain (TM).

**Figure 5:**
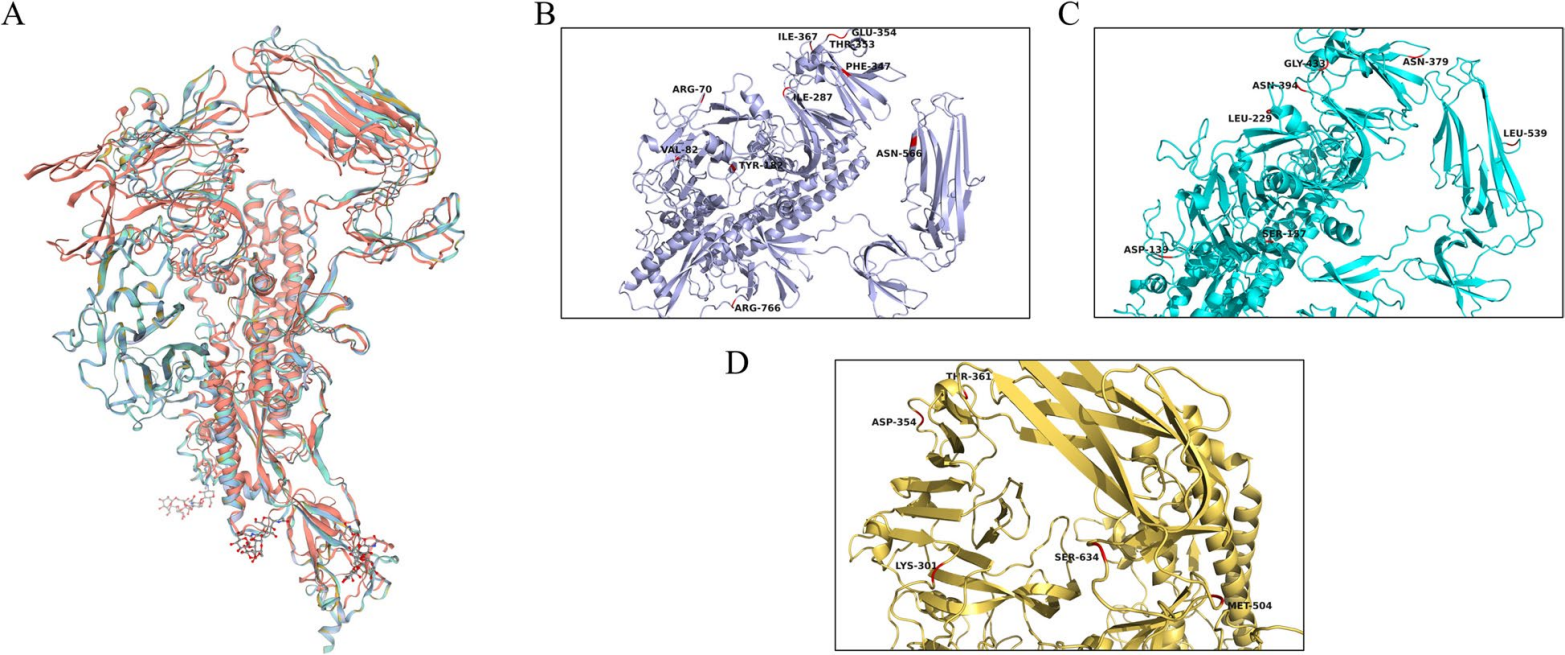
**Structural analysis of PEDV S protein**. **A** Comparative analysis of S protein monomer structure between PEDV-WF/2023, PEDV-SX/2024 and PEDV-HS/2024 strains and CV777 and PT-P5 strains. The S protein of PEDV-WF/2023, PEDV-SX/2024, PEDV-HS/2024, CV777 and PT-P5 strains cartoon modeling is shown as purple, light blue, yellow, red and blue, respectively. **B** Visualization of PEDV-WF/2023 amino acid mutations. **C** Visualization of PEDV-SX/2024 amino acid mutations. **D** Visualization of PEDV-HS/2024 amino acid mutations. Each strain in A.B.C.D is colored in the same scheme.

The amino acid mutation sites were analyzed based on the S gene, as shown in section “Phylogenetic analysis of coronavirus subfamily”, to further understand whether these mutated amino acids caused the changes in the tertiary structure of S protein. Therefore, the S protein structure of the three PEDV strains was compared with that of CV777 and PT-P5 strains (Figure 5A). The 3D structure of the three isolates, as well as CV777, showed significant changes in protein conformation in the D0 domain and the S1-NTD region, while the other regions were highly superimposed, although they all differed to different degrees. Next, we visualized the S-protein amino acid mutation sites of each strain. In the D0 region, PEDV-WF/2023 exhibited 3-aa mutations (Q70R, L82V, H182Y) compared with PT-P5, and PEDV-SX/2024 also exhibited three unique aa mutations (N139D, H157S, and P229L) (Figures 5B, C, and D). The different positions of the S1-NTD domains of PEDV-WF/2023, PEDV-SX/2024 and PEDV-HS/2024 were 5-aa, 3-aa and 3-aa, respectively. In the COE epitope region, PEDV-WF/2023, PEDV-SX/2024 and PEDV-HS/2024 had 1-aa (K566N), 1-aa (F539L) and 2-aa (L504M, P634S), respectively. PEDV-WF/2023 also had a 1-aa mutation (P766R) in the SS6 epitope region. Further studies are required to investigate whether the mutation of these amino acids and the change of protein tertiary structure have an impact on the virulence and pathogenicity of PEDV.

**Figure 6:**
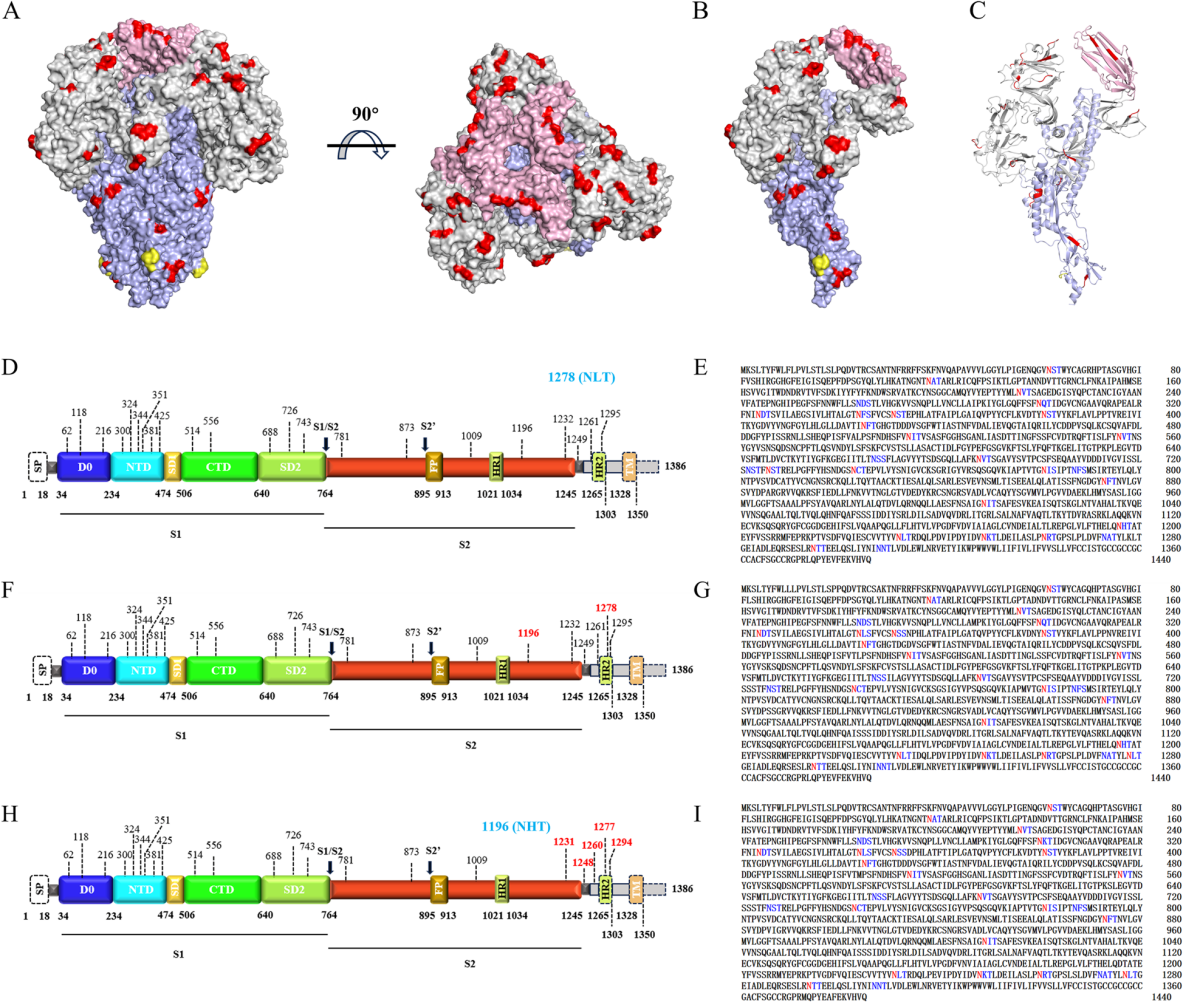
**The N-glycosylation structure maps of S protein in three PEDV strains obtained in this study. A** Atomic model of S protein trimer. **B** S protein monomer atomic model. **C.** Cartoon model of S protein monomer. N-glycosylation sites are shown in red. The lost N-glycosylation sites are indicated in yellow. The pink represents the receptor binding domain (RBD). The S1 and S2 subunits are shown in gray and purple, respectively. **D.** Distribution of N-glycosylation sites in the genome of PEDV-WF/2023 strain. **E.** Prediction results of N-glycosylation of PEDV-WF/2023 strain. **F** Distribution of N-glycosylation sites in the genome of PEDV-SX/2024 strain. **G** Prediction of N-glycosylation of PEDV-SX/2024 strain. **H** Distribution of N-glycosylation sites in genome of PEDV-HS/2024 strain.** I** PEDV-HS/2024 strain N-glycosylation prediction results. **D**, **F**, **H** in blue represent lost N-glycosylation sites. **E**, **G**, **I** Represent the results of specific tripeptide sequence outputs in blue and N-glycosylation modifications in red.

This study further predicted the N-glycosylation site of S protein. According to the modeling of the three isolates using SWISS-MODEL trimer and monomer structures were formed, and N-glycosylation map was extensively drawn on the surface of S protein (Figures 6A, B). It facilitated a better understanding of the relationship of N-glycosylation in S protein with protein structure and function. N-glycosylation of S protein mainly occurred in the α-helix and β-sheet, and wrapped tightly with them (Figure 6C). Through prediction, there were six dense N-glycosylation sites in the S1-NTD domain. PEDV-WF/2023 and PEDV-HS/2024 had a total of 22 N-glycosylation sites, of which 14 were located in S1 subunit and 8 in S2 subunit (Figures 6D, H). Compared with PT-P5, an N-glycosylation site was lost at position 1278 (NLT) and 1196 (NHT), respectively (Figures 6E, I). There were 23 N-glycosylation sites in PEDV-SX/2024, 14 of which were located in S1 subunit and 9 in S2 subunit (Figure 6F), which were consistent with the N-glycosylation sites of PT-P5 (Figure 6G).

### Pathogenicity analyses of the three isolated strains

**Figure 7:**
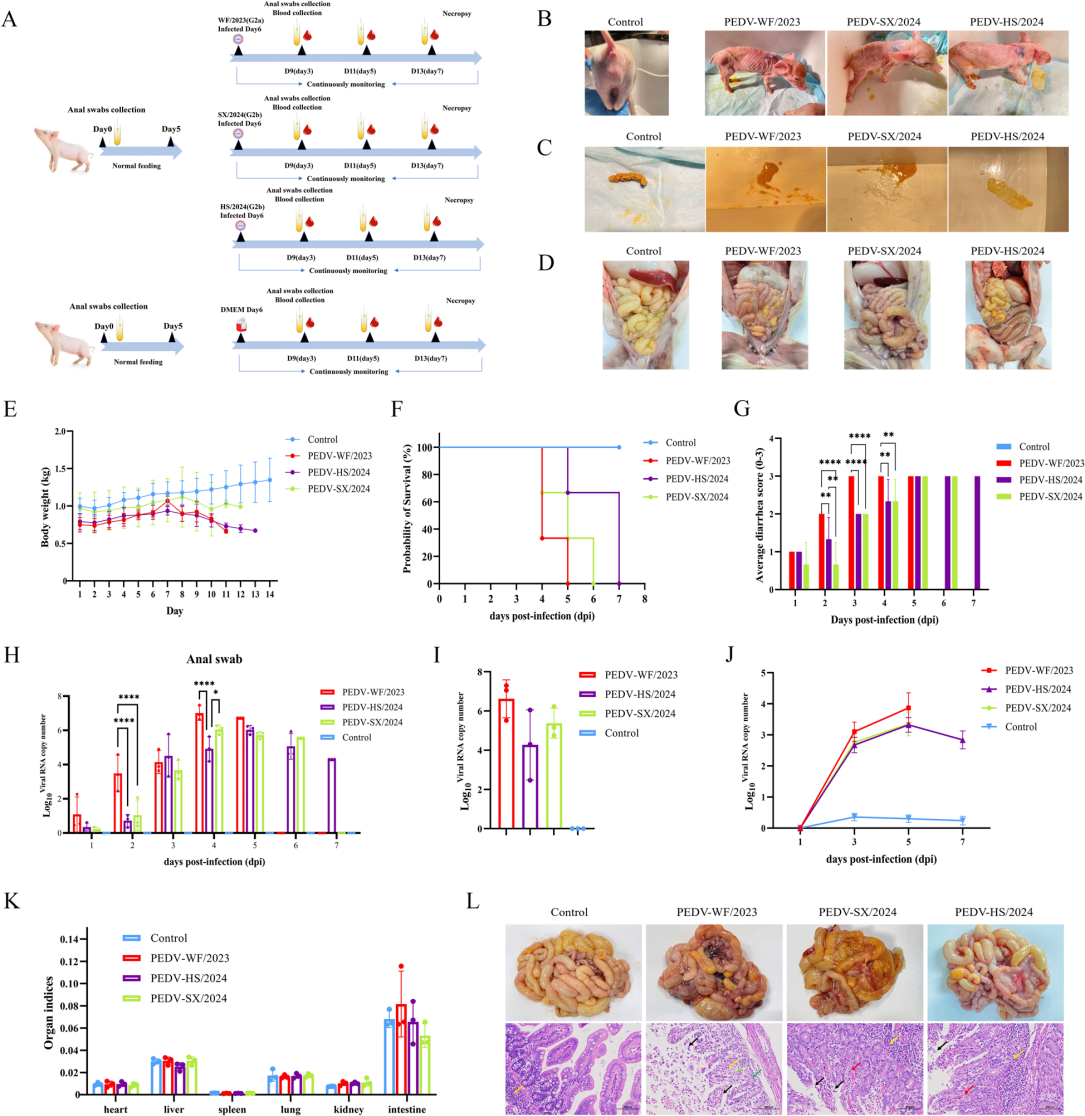
**Pathogenicity test of piglets with PEDV-WF/2023, PEDV-SX/2024 and PEDV-HS/2024 strains**.** A** Experimental challenge program for piglets. **B** Representative clinical signs of piglets in each group. **C** Fecal morphology evaluation of piglets in each group. **D** Gross lesions of the small intestine of piglets in each group. **E** Weight monitoring of piglets. **F** Survival rate of piglets. **G.**Piglet fecal scores. **H** Detection of viral RNA in anal swabs of piglets by RT-qPCR. *, *P* < 0.05; **, *P* < 0.01; ***, *P* < 0.001; ****, *P* < 0.0001. **I.** Viral load in jejunal tissues of piglets in different groups. **J** Viral load in blood of piglets in different groups. **K** Organ index of piglets in each group. **L** Histopathological examination and H&E staining of jejunum of piglets in PEDV challenge group or control group. Black arrow: mucosal epithelial cells necrosis, shedding; Red arrow: blood vessel congestion; Yellow arrow: focal aggregation of lymphocytes; Green arrow: intestinal gland; Light green arrow: connective tissue of the lamina propria of the intestinal villi; Scale bar, 100 μm.

A piglet pathogenicity test was designed to understand the harm of the three isolates on piglets, as shown in Figure 7A. After infection with PEDV in the three experimental groups, piglets in each group showed body wasting, vomiting and watery diarrhea to varied degrees (Figures 7B, C). PEDV-WF/2023 infected piglets also had bloody stools, and more severe diarrhea symptoms than those in the other two groups. Further necropsy of piglets after euthanasia in each group showed (Figure 7D) that the small intestine tissues of PEDV-infected piglets presented with thinning intestinal wall, transparency, flatulence and mesenteric bleeding, but those in the control group was normal and had no changes.

Compared with the control group, the three challenge groups had decreased weight of piglets to different degrees, particularly in the PEDV-WF/2023 group (Figure 7E). Piglets in the PEDV-WF/2023 group started to die at 4 dpi until all piglets died at 5 dpi, whereas piglets in the PEDV-HS/2024 group appeared to die the latest and at the slowest rate (Figure 7F). As shown in Figure 7G, piglets in PEDV-WF/2023 and PEDV-SX/2024 groups showed paste feces at 1 dpi, and those in PEDV-WF/2023 group showed significant differences compared with the other two groups at 2 dpi, 3 dpi and 4 dpi, and the diarrhea was the most severe at 3 dpi, 4 dpi and 5 dpi. None of the control piglets showed significant diarrhea symptoms throughout the experimental period (diarrhea score = 0). Moreover, at 2 dpi and 4 dpi, the detected virus shedding was significantly higher in group PEDV-WF/2023 than that in the other two groups, with the virus detoxification volume in this group reaching the highest at 4 dpi (Figure 7H). It was consistent with the time when the most severe diarrhea symptoms were observed in piglets in PEDV-WF/2023 group. In addition, the results of viraemia and viral load in the jejunum were consistent with those of anal swab analysis. In particular, the highest viral loads were found in small intestinal tissues of piglets in PEDV-WF/2023 group, whereas the lowest viral loads were found in PEDV-HS/2024 group (Figure 7I). At 5 dpi, the viral loads of the three PEDV strains in the blood all reached the peak, with the highest value in PEDV-WF/2023 group, which corresponded to the detection results of the viral load in intestinal tissue (Figure 7J). In addition, there was no obvious increase in the organ index (heart, liver, spleen, lung, kidney and intestine) of piglets in all groups with no significant differences (*P* > 0.05) (Figure 7K).

### Histopathological analysis

According to the results, extensive erosion was visible in the jejunal tissue of piglets in PEDV-WF/2023 group, with the observation of a large number of necrotic and shed mucosal epithelial cells, loosely arranged the lamina propria connective tissues and intestinal glands, and scattered distribution of a small number of lymphocytes. In PEDV-SX/2024 group, jejunal tissues of piglets were mainly observed with necrosis and shedding of mucosal epithelial cells, a small amount of vascular congestion, and more focal accumulation of lymphocytes. Furthermore, in PEDV-HS/2024 group, jejunal tissue of piglets showed a small amount of mucosal epithelial cell detachment, occasional vascular stasis, and lymphocyte aggregation. In the control group, the intestinal villi had normal morphological structure, and no obvious tissue lesions. Through comparison, PEDV-WF/2023 caused the most severe damage to the small intestinal tissue of piglets (Figure 7L). Therefore, PEDV-WF/2023 strain was confirmed to be the most pathogenic and most harmful epidemic strain to piglets.

## Discussion

Coronavirus PEDV poses a serious threat to swine populations in recent years, and has attracted widespread attention globally due to its the increased genetic diversity [32], leading to the complexity of viral transmission and mutation [9]. It shows higher infectivity and case fatality, posing a great challenge to the sustainable development of the entire breeding industry [33, 34]. At present, to control PEDV outbreaks and reduce piglet deaths, the fundamental issues lie in the establishment of strict biosafety, vaccination and feedback [35, 36]. Before 2010, PEDV was identified to be almost a classic G1 strain with slow mutation rate in China. At that time, our inactivated PEDV vaccine and attenuated vaccine still had good protective effect against PEDV mutant strains [37]. However, the protective effect of these vaccines became insufficient with the intensification of PEDV genetic variation after 2010 [17, 32]. Nowadays, attenuated vaccines based on the G2b subtype AJ1102 strain are widely used, and there are many types of commercial vaccines. Nevertheless, it remains a great obstacle to prevent and control PEDV in our country. Therefore, it is particularly important to understand the molecular structure characteristics, evolution trend, transmission dynamics and pathogenicity of current circulating strains.

It is currently a difficulty to realize in vitro isolation of PEDV [38]. In this study, three PEDV strains were successfully isolated from diarrhea samples and diseased intestinal tissues in North China using Vero-CCL-81 cells. Trypsin can significantly promote the effectiveness of the virus intracellularly considering its importance in PEDV infection and isolation [39, 40]. In this study, trypsin at a final concentration of 7 μg/mL was used to improve the efficiency of virus infection of host cells. Typical CPE appeared at F5, with significant increase in PEDV mRNA levels, similar to previous research [31]. Some studies have also confirmed that IFA technology has advantages in the detection of virus antigens [41]. Therefore, this study successfully detected PEDV antigens in the cytoplasm of PEDV-infected cells by IFA verification technology. Our subsequent study focused on the analysis of the phylogenetic and evolution trend of the three isolates to grasp the genome variation of the current epidemic strains in China.

Frequent mutations in PEDV gene induce significantly adverse impacts on global pig industry [42], affecting the economy, supply chain and vaccine effectiveness. Amino acid mutation, deletion and genetic recombination are important mechanisms for virus evolution [43]. Especially for coronaviruses, these mutations may alter viral characteristics (e.g., increased transmissibility, pathogenicity, or evasion of antiviral drugs and host immune responses), thus posing a potential threat to the life and health of humans and other species. Based on the phylogenetic tree of different coronavirus sequences of the S gene, the PEDV strain we studied belongs to the Alpha-coronavirus genus together with BtCoV/512/2005, bat coronavirus HKU8 (BtCoV HKU8 AFCD77), human coronavirus 229 (HCoV-229) and TGEV. SARS-CoV and SARS-CoV-2 belong to the genus Beta-coronavirus. More specifically, the PEDV strain belongs to the same branch as the bat coronavirus (BtCoV/512/2005) from Hong Kong, China, both of which have the closest relationship. PEDV has been reported to have highly similar genome sequences to coronaviruses in bats [9, 44, 45]. The recently emerged swine acute diarrhea syndrome coronavirus (SADS-CoV) is transmitted from bats to pigs [46]. Moreover, bats are also thought to be the natural hosts of human coronaviruses such as SARS-CoV and HCoV-229. PEDV can also recognize the human aminopeptidase N receptor and infect cells from humans, monkeys and bats [44]. Therefore, bats may be the origin of the evolution of Alpha-coronavirus and Beta-coronavirus [2], and PEDV may also infect other species (especially humans).

There is an urgent need to uncover the phylogenetic and molecular structure characteristics of PEDV epidemic strains, especially the S and ORF3 genes, both of which affect the virulence of PEDV [47]. In accordance with the constructed phylogenetic tree based on the S and ORF3 genes, PEDV strains were mainly divided into two genotypes of G1 (classic type) and G2 (variant type). Each genotype has two subtypes of G1a and G1b, as well as G2a and G2b. In this study, PEDV-WF/2023 is a G2a subtype, and PEDV-SX/2024 and PEDV-HS/2024 are G2b subtypes. At present, G2 genotype (89.9%) is still the dominant epidemic strain in China [48]. Indirectly, the three PEDV G2 genotype strains isolates in this study can basically represent the current epidemic strains in China. Furthermore, based on the homology of S and ORF3 genes, the amino acid homology between G2a and G2b subtypes was as high as 97–98%, showing atypical amino acid differences between them. It may suggest a relatively small the genetic difference between different genotypes in the S gene. The proposed high homology may affect the development and effectiveness of vaccines, mask potential mutations of the virus, and thus produce negative impact on the identification of epidemiological characteristics [49]. In contrast, the ORF3 gene achieved more effective distinguishing between G2a and G2b subtypes. The similarity of ORF3 gene was only 95–96% between G2a and G2b subunits. Moreover, 25aa, 80aa, 107aa, 168aa and 182aa were all representative mutation sites of G2a and G2b subtypes, resulting in obvious sequence differences in the ORF3 genes of the two subtypes. These differences may affect the ability of the virus to replicate and its ability to escape the host immune system [50]. In general, the G1b subtype strain is a low-pathogenic genotype virus of PEDV [51]. Its mortality rate is usually significantly lower than that of G1a, G2a and G2b, despite its infection rate reaching a certain level [52]. In this study, there were 133 aa deletions in the ORF3 gene of the G1b subtype from 92 to 224aa, which may affect the pathogenicity of the virus. In the past, deletion and truncation of the ORF3 protein of PEDV strains have been documented to reduce the pathogenicity in piglets [51]. Lu et al. [50] found that the use of G2b 17GXCZ-1ORF3d-P120 vaccine in immunized sows significantly increased the levels of PEDV-specific IgG and IgA antibodies in piglets. It may indicate that attenuated strains with truncated ORF3 genes may be promising candidate vaccines for preventing and controlling PEDV, also offering a new perspective for the molecular epidemiology of PEDV.

The S protein is considered to be the main pathogenic protein of PEDV, and its structure can directly affect the pathogenicity and immune evasion ability of the virus [11]. Compared with CV777 and PT-P5, pI values of PEDV-WF/2023, PEDV-SX/2024 and PEDV-HS/2024 were 5.35, 5.19 and 5.21, respectively, which were similar to that of PT-P5, while the pI value of CV777 was 5.11. The changes in these values may suggest potential differences in the amino acid composition of different virus strains. At PH below the isoelectric point, S protein may be positively charged and bind more easily to negatively charged cell membranes, thus facilitating viral invasion [53]. Furthermore, the aliphatic index of S protein exhibits an intimate association with its function in the cell membrane. Jia et al. [54] revealed an aliphatic index of PEDV S protein of 93.21, supporting its stability and affinity in a lipid environment. In this study, the aliphatic indexes of the three PEDV strains were all above 90. A high aliphatic index may suggest that the S protein of these strains has strong integration in the membrane structure, which can promote the invasion of the virus and improve the overall infection ability of the virus [55]. In addition, the instability index of the S protein of the three strains was around 31. Theoretically, the protein is judged to be stable if the instability index is below 40. Combining the lipid solubility and stability of the S protein, the S protein of the PEDV strain obtained in this study was speculated to maintain its function on the cell membrane, thereby effectively executing the infection mechanism of the virus [54].

Furthermore, the S protein is the most important structural protein on the surface of virus particles. Based on the analysis of the secondary structure of the S protein, compared with PT-P5, in the COE region of the S protein, PEDV-SX/2024 had a β-sheet deletion at 330aa–332aa; while PEDV-WF/2023 and PEDV-SX/2024 lost an identical at 402aa–403aa. At 453aa, a new α-helix structure appeared in PEDV-WF/2023. No significant spatial conformational changes were observed in the 3D structural model of the S protein, possibly due to the shorter amino acids encoded by the altered secondary structure. However, these α-helix and β-sheet changes might have an impact on the protein's stability and ability to bind to host cell surface receptors [8, 56]. The S protein exists as a trimer, with each monomer containing a D0 domain. The D0 domain is an additional domain at the N terminal of the S1 subunit of PEDV S protein, which is a unique feature of Alpha-coronavirus and can preferentially bind sialic acid [11]. For further clarification of differences in the tertiary structure of S protein, the spatial conformation of D0 domain of the three isolates was significantly different from that of CV777. By comparison, the D0 region of the S protein of PEDV CV777 was upward and existed in a D0-up state, which was similar to the S protein conformation of some other coronaviruses (e.g., feline infectious peritonitis virus FIPV) [13]. This conformation has been proven to enable the optimization of the binding to sialic acid receptors through its specific spatial structure and charge distribution [57]. Relatively speaking, the D0 domain of the three PEDV strains was similar to that of PT-P5 strain, and was opposite to the D0-up conformation, showing a downward direction. The D0 region of this conformation may be obscured, which is associated with some functional disorders of the virus [57]. The difference in the conformation of the D0 domain may affect the transmission and infection mechanism of the virus [11]. Inspired by this, it may be feasible to simulate the characteristics of the D0-up conformation to enhance the immune response, thereby improving the prevention of PEDV infection [58].

In general, S protein is considered to be a structural protein with the most diverse genetic evolution and the fastest mutation frequency [59]. Mutations of S protein in different PEDV strains may promote the production of different binding capabilities with sialic acid by the D0 domain [60]. In this study, compared with CV777 and PT-P5, PEDV-WF/2023 had three unique aa mutation sites, namely Q70R, L82V and H182Y, in the D0 domain (34aa–234aa). There were also three unique aa mutation sites (N139D, H157S, P229L) in PEDV-SX/2024. The D0 domain is a key target for the host antibody response, its mutation may therefore offer a pathway for immune escape from PEDV, resulting in antibodies not being able to effectively recognize the region, and eventually reduced protective effectiveness of the vaccine [57]. As indicated by existing data, there were significant aa mutations between different PEDV genotypes, which were mainly concentrated in the N-terminal domain of S protein (S1-NTD) [61, 62]. This view was also confirmed taking into consideration of the mutation sites of the three PEDV strains. PEDV-WF/2023 was observed to have 5 aa mutations (M287I, L345F, S353T, N354E and T367I) in the S1-NTD region; while three aa mutations were found in PEDV-SX/2024 (T379N, T394N, D433G) and PEDV-HS/2024 (Q301K, N354D, A361T), respectively. Key mutations in the N-terminal domain can give viruses a competitive advantage in co-infection with other viruses [63]. It is not difficult to interpret that the S1-NTD may be an important region to unveil the genetic relationship of different genotypes. Moreover, the mutation of amino acids in antigenic epitopes may also alter the immunogenicity of the virus [64]. In our study, there were unique aa mutations in COE epitope regions (504aa–638aa) and SS6 (764aa–771aa) of these three PEDV strains, showing genetic diversity. Mutations in these regions are closely related to antibody binding [65], and the cell attachment domain of the S1 subunit is the main target of neutralizing antibodies. Consequently, changes in these targets will also affect both the binding ability and neutralizing effect of the antibody [66]. PEDV S protein is also a protein with intensive N-glycosylation, especially in the S1-NTD region [67], and mutations in this region may also affect the deletion and generation of N-glycosylation sites [68]. According to our prediction of N-glycosylation sites, the three PEDV strains all clustered 6 N-glycosylation sites in the S1-NTD region. Compared with PT-P5, PEDV-WF/2023 lost an N-glycosylation site at 1278 (NLT), and PEDV-HS/2024 had an N-glycosylation site missing at 1196 (NHT), while the N-glycosylation site of the PEDV-SX/2024 was completely consistent with that of PT-P5. Changes in N-glycosylation sites may directly affect the pathogenicity of viruses [11]. In addition, the highly glycosylated S protein can reduce the recognition and clearance opportunity of the host immune system by masking key immune epitopes, and affect the effectiveness of the vaccine and the transmission ability of the virus [13]. Therefore, in order to boost vaccine development, it is necessary to elucidate the molecular structure characteristics and evolution trends of the current circulating strains in China. Moreover, further in-depth investigation is needed to reveal the specific effects of these mutations on the function and virulence of PEDV.

It is well known that PEDV G1 genotype is relatively early, has a long evolutionary distance, with relatively mild clinical symptoms caused by the infection are relatively mild [48]. In contrast, G2 genotype, with genetic diversity and frequent mutation, is significantly more infectious and pathogenic than G1 genotype, leading to potentially higher proportion of death and more severe clinical symptoms [8, 43]. Moreover, the G2a subtype is a newer mutant strain than the G2b subtype, causing increased frequency of epidemic outbreaks in recent years [50]. The present study compared the pathogenicity of the G2a and G2b subtypes of the G2 genotype through animal experiments. Piglets infected with the three PEDV strains all showed different degrees of diarrhea, vomiting, anorexia and body wasting symptoms. Piglets in the PEDV-WF/2023 group had severe diarrhea accompanied by bloody stool. In terms of the possible reason, after infection in these experimental piglets, the virus attacks the intestinal epithelial cells, causing the intestinal villi to atrophy and fuse, affecting intestinal absorption, resulting in severe diarrhea and dehydration [69]. Macroscopic autopsy also revealed dilation of the intestinal lumen, thinning of the intestinal wall, and mesenteric bleeding in the small intestine of infected piglets. Further histopathological analysis showed that in the small intestinal tissue of piglets in the PEDV-WF/2023 group, it was observed with extensive erosion, a large number of mucosal epithelial cells, necrotic and shed intestinal gland epithelial cells, and a large area of connective tissue and intestinal glands in the lamina propria with loose arrangement. Clearly, the damage to small intestinal tissue was the most severe in this group, different from the other two groups. PEDV has been reported to induce cell dysfunction and death through binding with small intestinal epithelial cells, thus destroying intestinal barrier function [70]. More importantly, piglets infected with G2a strain were found to have the fastest decrease in the weight, the earliest occurrence of diarrhea and death, and all piglets died at 5 dpi. The level of viral RNA shed from piglet feces peaked when infected with G2a strain at 4 dpi. In addition, due to the tissue tropism of the PEDV pathogen, its main target organ is the small intestine [71]. This study also detected the viral load in the diseased tissues of the small intestine and in the blood. The viral load of G2a strain was higher than that of G2b strain in both the small intestine tissue and the blood, and the viral RNA content in the small intestine tissue was higher than that in the blood, further confirming the aforementioned view.

In summary, this study isolates one G2a strain PEDV-WF/2023 and two G2b strains PEDV-SX/2024 and PEDV-HS/2024 from the small intestinal diseased tissue successfully, combined with further analyses of their genetic characteristics and pathogenicity. We acquire additional knowledge on the physicochemical properties, tertiary structure, molecular evolutionary features and differences in pathogenicity of PEDV strains of different genotypes. It may facilitate our understanding of the structural characteristics, evolution trend and transmission dynamics of the currently prevalent strains. More targeted strategies for the prevention and control of PEDV can be developed based on the unique molecular characteristics of different genotypes. Findings in this study may provide potential reference for formulating effective prevention and control strategies, and theoretical basis for developing new vaccines and treatment options to curb the cross-species spread of PEDV in pigs and other species (especially humans).

## Supplementary Information

**Additional file 1: The GenBank accession numbers for reference Coronaviruses strains.**

**Additional file 2: The GenBank accession numbers for reference PEDV strains S gene.**

**Additional file 3: The GenBank accession numbers for reference PEDV strains ORF3 gene.**

## Data Availability

All data generated or analyzed during this study are included in this published article [and its supplementary information files].
