# Supplementary material for: Genetic characterization and pathogenicity analysis of three porcine epidemic diarrhea virus strains isolated from North China
Source: Vet Res. 2025 Jun 14;56:118. doi: 10.1186/s13567-025-01554-4 (PMC12166606; doi:10.1186/s13567-025-01554-4)
Supplement: Supplementary file 1 — Additional file 1: The GenBank accession numbers for reference Coronaviruses strains. [file 13567_2025_1554_MOESM1_ESM.docx]

**Additional file 1 The GenBank accession numbers for reference Coronaviruses strains**

| **strains** | **genus** | **GenBank accession No.** | |  |
| --- | --- | --- | --- | --- |
| Human coronavirus 229 | Alphacoronavirus | | AF304460.1 | |
| CV777 | Alphacoronavirus | | AF353511.1 | |
| AJ1102 | Alphacoronavirus | | JX188454.1 | |
| AH2012 | Alphacoronavirus | | KC210145.1 | |
| AH2012/12 | Alphacoronavirus | | KU646831.1 | |
| TGEV Miller M6 | Alphacoronavirus | | DQ811785.1 | |
| Bat coronavirus HKU8 AFCD77 | Alphacoronavirus | | EU420139.1 | |
| BtCoV/512/2005 | Alphacoronavirus | | DQ648858.1 | |
| Bat SARS-like Rs4084 | Betacoronavirus | | KY417144.1 | |
| BCoV-ENT | Betacoronavirus | | AF391541.1 | |
| BCoV Mebus | Betacoronavirus | | U00735.2 | |
| BCoV Kakegawa | Betacoronavirus | | AB354579.1 | |
| Human betacoronavirus 2c EMC/2012 | Betacoronavirus | | JX869059.2 | |
| SARS TW7 | Betacoronavirus | | AY502930.1 | |
| SARS-CoV-2/human/CHN/IME-HZ01/2020 | Betacoronavirus | | MT039874.1 | |
| Tylonycteris bat coronavirus HKU4 | Betacoronavirus | | EF065505.1 | |
| Porcine deltacoronavirus 8734/USA-IA/2014 | Deltacoronavirus | | KJ567050.1 | |
| Quail deltacoronavirus G032/2015 | Deltacoronavirus | | MH532440.1 | |
| White-eye coronavirus HKU16 | Deltacoronavirus | | NC_016991.1 | |
| Porcine deltacoronavirus HB-BD | Deltacoronavirus | | MF948005.1 | |
| Avian infectious bronchitis virus | Gammacoronavirus | | NC_001451.1 | |
| Beluga Whale coronavirus SW1 | Gammacoronavirus | | NC_010646.1 | |
| Infectious bronchitis virus Sczy3 | Gammacoronavirus | | JF732903.1 | |
