# Supplementary material for: Genetic characterization and pathogenicity analysis of three porcine epidemic diarrhea virus strains isolated from North China
Source: Vet Res. 2025 Jun 14;56:118. doi: 10.1186/s13567-025-01554-4 (PMC12166606; doi:10.1186/s13567-025-01554-4)
Supplement: Supplementary file 2 — Additional file 2: The GenBank accession numbers for reference PEDV strains S gene. [file 13567_2025_1554_MOESM2_ESM.docx]

**Additional file 2 The GenBank accession numbers for reference PEDV strains S gene**

| **strains** | **subtribe** | **GenBank accession No.** |
| --- | --- | --- |
| CV777 | G1a | AF353511.1 |
| DR13 | G1a | DQ862099.1 |
| LZC | G1a | EF185992.1 |
| SM98 | G1a | GU937797.1 |
| CHM2013 | G1a | KM887144.1 |
| AVCT12 | G1a | LC053455.1 |
| CV777 | G1b | KT323979.1 |
| DR13 | G1b | JQ023162.1 |
| SD-M | G1b | JX560761.1 |
| SC1402 | G1b | KP162057.1 |
| SQ2014 | G1b | KP728470.1 |
| USA/Indiana12.83/2013 | S-INDEL | KJ645635.1 |
| CH/GD-06/2012 | S-INDEL | KP870118.1 |
| CH-ZWC-01-2015 | S-INDEL | KR296682.1 |
| CZ2015 | S-INDEL | KY381581.1 |
| CH/FJXM-2/2012 | S-INDEL | JX070672.1 |
| CH/HBQX/10 | S-INDEL | JX501318.1 |
| OH851 | S-INDEL | KJ399978.1 |
| CH/ZMDZY/11 | G2a | KC196276.1 |
| AH2012 | G2a | KC210145.1 |
| JS-HZ2012 | G2a | KC210147.1 |
| KNU-1305 | G2a | KJ451040.1 |
| USA/Minnesota79/2013 | G2a | KJ645674.1 |
| USA/Iowa96/2013 | G2a | KJ645688.1 |
| CPGEN 20140427 | G2a | KJ777678.1 |
| XJ-DB2 | G2a | KM287429.1 |
| ZJU/G2/2013 | G2a | KU558701.1 |
| KNU-1601 | G2a | KY963963.1 |
| MYG-1/JPN/2014 | G2a | LC063838.1 |
| GDS22 | G2a | MH726368.1 |
| GDS01 | G2b | AB857233.1 |
| CHGD-01 | G2b | JN980698.1 |
| AJ1102 | G2b | JX188454.1 |
| LC | G2b | JX489155.1 |
| ZJCZ4 | G2b | JX524137.1 |
| YN1 | G2b | KT021227.1 |
| AH2012/12 | G2b | KU646831.1 |
| CH/JLDH/2016 | G2b | MF346935.1 |
| LW/L | G2b | MK392335.1 |
| YN150 | G2b | MZ581326.1 |
| LZ202401 | G2b | PP373526.1 |
