# Supplementary material for: Genetic characterization and pathogenicity analysis of three porcine epidemic diarrhea virus strains isolated from North China
Source: Vet Res. 2025 Jun 14;56:118. doi: 10.1186/s13567-025-01554-4 (PMC12166606; doi:10.1186/s13567-025-01554-4)
Supplement: Supplementary file 3 — Additional file 3: The GenBank accession numbers for reference PEDV strains ORF3 gene. [file 13567_2025_1554_MOESM3_ESM.docx]

**Additional file 3** **The GenBank accession numbers for reference PEDV strains ORF3 gene.**

| **strains** | **subtribe** | **GenBank accession No.** |
| --- | --- | --- |
| CV777 | G1a | AF353511.1 |
| LZC | G1a | EF185992.1 |
| SM98 | G1a | GU937797.1 |
| CHM2013 | G1a | KM887144.1 |
| CV777 | G1b | KT323979.1 |
| DR13 | G1b | JQ023162.1 |
| SD-M | G1b | JX560761.1 |
| SC1402 | G1b | KP162057.1 |
| SQ2014 | G1b | KP728470.1 |
| USA/Indiana12.83/2013 | S-INDEL | KJ645635.1 |
| USA/Iowa106/2013 | S-INDEL | KJ645695.1 |
| USA/IA/2013/19321 | S-INDEL | KM975738.1 |
| Hawaii/39249/2014 | S-INDEL | KP688354.1 |
| USA/Minnesota52/2013 | S-INDEL | KJ645704.1 |
| OH851 | S-INDEL | KJ399978.1 |
| CH/ZMDZY/11 | G2a | KC196276.1 |
| AH2012 | G2a | KC210145.1 |
| JS-HZ2012 | G2a | KC210147.1 |
| KNU-1305 | G2a | KJ662670.1 |
| USA/Minnesota79/2013 | G2a | KJ645674.1 |
| USA/Iowa96/2013 | G2a | KJ645688.1 |
| LZW | G2a | KJ777678.1 |
| XJ-DB2 | G2a | KM386647.1 |
| ZJU/G2/2013 | G2a | KU558701.1 |
| TC-PC177 | G2a | KY499261.1 |
| KNU-1601 | G2a | KY963963.1 |
| GDS22 | G2a | MH726368.1 |
| GDS01 | G2b | KM089829.1 |
| CHGD-01 | G2b | JX261936.1 |
| AJ1102 | G2b | JX188454.1 |
| LC | G2b | JX489155.1 |
| ZJCZ4 | G2b | JX524137.1 |
| YN1 | G2b | KT021227.1 |
| AH2012/12 | G2b | KU646831.1 |
| CH/JLDH/2016 | G2b | MF346935.1 |
| LW/L | G2b | MK392335.1 |
| YN150 | G2b | MZ581326.1 |
